# Electron correlation in Li^+^, He, H^−^ and the critical nuclear charge system *Z*_*C*_: energies, densities and Coulomb holes

**DOI:** 10.1098/rsos.181357

**Published:** 2019-01-09

**Authors:** Adam L. Baskerville, Andrew W. King, Hazel Cox

**Affiliations:** Department of Chemistry, School of Life Sciences, University of Sussex, Falmer, Brighton BN1 9QJ, UK

**Keywords:** electron correlation, Coulomb hole, intracule density, two-electron systems, critical nuclear charge for binding

## Abstract

This paper presents high-accuracy correlation energies, intracule densities and Coulomb hole(s) for the lithium cation, helium, hydride ion and the system with the critical nuclear charge, *Z*_*C*_, for binding two electrons. The fully correlated (FC) wave function and the Hartree–Fock (HF) wave function are both determined using a Laguerre-based wave function. It is found that for the lithium cation and the helium atom a secondary Coulomb hole is present, in agreement with a previous literature finding, confirming a counterintuitive conclusion that electron correlation can act to bring distant electrons closer together. However, no evidence for a tertiary Coulomb hole is found. For the hydride anion and the system just prior to electron detachment only a single Coulomb hole is present and electron correlation decreases the probability of finding the electrons closer together at all radial distances. The emergence of a secondary Coulomb hole is investigated and found to occur between *Z* = 1.15 and *Z* = 1.20. The FC and HF energies and intracule densities (in atomic units) used to calculate the correlation energy and Coulomb hole, respectively, are accurate to at least the nano-scale for helium and the cation and at least the micro-scale for the anions.

## 1. Introduction

Understanding and accurately modelling electron correlation is essential for interpreting chemical processes. High-accuracy correlation data are therefore vital for the development and/or testing of new quantum chemistry methods. The two-electron atom or ion (such as He and its isoelectronic sequence) is the smallest system that requires a full treatment of electron correlation. Hylleraas established the importance of including the electron–electron distance (*r*_12_) in the coordinate system [1,2], and there has been a wealth of accurate calculations since [3–10].

However, extending this fully correlated (FC) approach beyond heliogenic systems comes at a high computational cost, and so mainstream computational quantum chemistry is built on the one-electron operators arising in the Hartree–Fock (HF) method for solving the many-electron Schrödinger equation. In fact, ‘correlation energy’ usually refers to the missing Coulomb correlation energy in the HF treatment *E*_corr_ defined as *E*_corr_ = *E* − *E*_*HF*_, where *E*_*HF*_ is an upper bound to *E*, the exact eigenvalue of the Hamiltonian for the state under consideration. Coulson & Neilson [11] calculated the distribution function of the inter-electronic distance *r*_12_ (often called the intracule distribution function) in the ground state of helium, and defined the Coulomb hole as the difference between the distribution function derived from their best approximation to the true wave function and from their best HF wave function. They investigated the size and depth of the Coulomb hole in helium and later investigated the change in the shape and size of the Coulomb hole with nuclear charge [12]. More recently, Pearson *et al.* [13] quantified the properties of the Coulomb hole for helium and helium-like cations. They showed that a second hole emerged as the quality of the basis for the HF calculation was improved. The maximum error in their intracule density was of the order of 10^−6^.

The aim of this paper is to determine electron correlation data to high precision, even for low nuclear charge anionic systems. Over a number of years, we have developed a suite of programs to calculate high-accuracy, fully correlated three-body energies and wave functions. The methodology used extends the original work of Pekeris on heliogenic systems [7]. Recently [14], we implemented Hartree–Fock theory using a similar choice of wave function and coordinate system, i.e. a Laguerre-basis in perimetric coordinates, in order to have complete control over the optimization of the HF wave function and with a view to provide a balanced treatment of electron correlation for all values of *Z*.

Particularly desirable is high-accuracy electron correlation data in the low-density regime for both rigorous testing of long-range behaviour in new methods and for the development of correlation functionals for use in density functional theory. To provide this low-density, long-range information, we determine electron correlation data for a two-electron system just prior to electron detachment: the critical nuclear charge system, *Z*_*C*_. Previously, we presented a variational method for calculating *Z*_*C*_, using the Laguerre-based series solution methodology in the present work and obtained a value of *Z*_*C*_ = 0.911028224 [15]. This value was in good agreement with the best literature value of 0.91102822407725573(4) obtained using Hylleraas coordinates [16]. Here we investigate the effect of correlation on bound state properties and determine correlation energies, intracule density distributions, and the properties of the Coulomb holes for Li^+^, He, H^−^ and the critical nuclear charge system *Z*_*C*_, to shed light on short-range and long-range correlation effects in two-electron systems.

## 2. Material and method

### 2.1. Hartree–Fock (HF) and fully correlated (FC) methodologies

The singlet ground state of helium and helium-like systems, within the clamped nucleus approximation, are investigated. The details of the methodology used for the FC systems are described in [17] and for HF systems in [14]. Atomic units are used throughout, i.e. $m_{e}=\hbar ={\left(4\pi \epsilon_{0}\right)}^{-1}=e=1$. All calculations use 32 digits of precision at every stage to ensure high accuracy. All HF data presented uses a 20-term wave function and all FC data presented uses a 4389-term wave function (see results for quality of energy and wave function).

In brief, a Laguerre-based wave function in scaled interparticle and/or perimetric coordinates is used. The Laguerre polynomials with an exponential weight factor, *e*^−*x*/2^, form an orthogonal set and are defined for the range [0, ∞). The perimetric coordinates *z*_*i*_ are linear combinations of the interparticle distances *r*_1_, *r*_2_ and *r*_12_ (sometime labelled *r*_3_), i.e. *z*_1_ = (*r*_2_ + *r*_12_ − *r*_1_), *z*_2_ = (*r*_12_ + *r*_1_ − *r*_2_), and *z*_3_ = (*r*_1_ + *r*_2_ − *r*_12_); they are independent and each ranges from 0 to ∞.

The HF wave function, *ψ*_HF_, is taken as the product

2.1 $$\psi_{\mathrm{HF}}(r_{1},r_{2})=\psi (r_{1})\psi (r_{2}),$$

where the required anti-symmetry of the total wave function is embedded in the spin part which has been integrated out. The *ψ*(*r*_*i*_) have the form

2.2 $$\psi (r_{i})=e^{-(1/2)Ar_{i}}\sum_{q=0}^{\infty }C(q)L_{q}(Ar_{i}),\;i=1\;\text{or}\;2,$$

and *r*_1_ and *r*_2_ are the nucleus–electron 1 and nucleus–electron 2 interparticle distances, respectively. *L*_*n*_(*x*) is a Laguerre polynomial of degree *n* and *A* is a nonlinear variational parameter introduced to increase the rate of convergence for a given basis set size and optimized using the quadratic interpolation method employed by the computer algebra program Maple [18].

The FC wave function, *ψ*_FC_, explicitly includes the electron–electron distance *r*_12_, in addition to the nucleus–electron distances *r*_1_ and *r*_2_, and takes the form

2.3 $$\psi_{\mathrm{FC}}(z_{1},z_{2},z_{3})=e^{-(1/2)(\alpha z_{1}+\alpha z_{2}+\gamma z_{3})}\sum_{l,m,n=0}^{\infty }A(l,m,n)L_{l}(\alpha z_{1})L_{m}(\alpha z_{2})L_{n}(\gamma z_{3}),$$

where *α* and *γ* are nonlinear variational parameters, such that when *γ* = 2*α* the exponent in the wave function models, in principle, the correct asymptotic behaviour of the solution of the Schrödinger equation for two-electron atoms at large *r*_1_ and *r*_2_ [1,3]. Both the 1-parameter wave function, where *γ* is constrained to equal 2*α*, and the 2-parameter wave function, where *γ* and *α* are varied independently, were tested (see electronic supplementary material). It was found that there is very little difference in the quality of the results, therefore, the computationally cheaper 1-parameter wave function results are presented in this paper. The wave function *ψ*_FC_ is substituted into the Schrödinger equation and the resulting generalized eigenvalue equation for the fully correlated system is solved using a series solution method described by Cox *et al.* [4,17] and based on the original work of Pekeris [3]. The standard Laguerre recursion relations are used to eliminate the powers and derivatives of the variables, resulting in a 33-term recursion relation between the coefficients *A*(*l*, *m*, *n*) in equation (2.3).

For the HF systems, the one-electron terms are amenable to series solution, resulting in a 5-term recursion relation between the coefficients *C*(*q*) in (2.2). The recursion relation represents a set of linear equations for the determination of the coefficients *C*(*q*), and the vanishing of their determinant yields the hydrogen-like *core* energy eigenvalues. These one-electron terms are very fast to calculate using the series solution method. For the two-electron integrals, explicit integration is required; however, these integrals can be solved analytically by exploiting the properties of the Laguerre polynomials after first converting to perimetric coordinates to give independent integration domains [14]. The sum of the one-electron and two-electron matrix elements are used to create the Fock matrix and the Fock equations are solved as a generalized eigenvalue problem, to determine new wave function coefficients. The convergence threshold for the self-consistent field (SCF) procedure was set at 3 × 10^−15^ and was performed using direct inversion of iterative space (DIIS).

### 2.2. Intracule distribution functions

The Coulomb hole is defined as the difference in the distribution function of the inter-electronic distance, *r*_12_, i.e. the intracule, for the correlated wave function and the Hartree–Fock wave function [11]. The intracule density is defined as

2.4 $$h(r)\equiv \rho_{12}(r)=\langle \psi |\delta (r_{12}-r)|\psi \rangle .$$

It measures the radial correlation between two like-charged particles where *r*_12_ is the distance between them. The intracule distribution function, *D*(*r*) = 4π*r*^2^*h*(*r*), is normalized to unity such that $4\pi \int_{0}^{\infty }r^{2}h(r)\;\text{d}r=1$. The difference between the intracule distribution functions generated with the statistically independent and uncorrelated HF approximation and the explicitly correlated FC method defines the Coulomb hole, using

2.5 $$\Delta D(r)=D_{\mathrm{FC}}(r)-D_{\mathrm{HF}}(r),$$

where Δ*D*(*r*) is calculated as the numerical difference between *D*_FC_(*r*) and *D*_HF_(*r*).

To provide an estimate of the intracule accuracy, we use the definition of the maximum error in the intracules that was employed by Pearson *et al.* [13]. The errors associated with the intracule densities are calculated using two forms. The first is the root mean square (RMS) error given by

2.6 $${\left(\int_{0}^{\infty }{\left[D_{\mathrm{FC}}^{4389}(r)-D_{\mathrm{FC}}^{2856}(r)\right]}^{2}\text{d}r\right)}^{1/2}\;\text{and}\;{\left(\int_{0}^{\infty }{\left[D_{\mathrm{HF}}^{20}(r)-D_{\mathrm{HF}}^{15}(r)\right]}^{2}\text{d}r\right)}^{1/2}$$

and the second is the maximum error,

2.7 $$\max_{r>0}|D_{\mathrm{FC}}^{4389}(r)-D_{\mathrm{FC}}^{2856}(r)|\;\text{and}\;\max_{r>0}|D_{\mathrm{HF}}^{20}(r)-D_{\mathrm{HF}}^{15}(r)|.$$

We interpret the values from (2.6) and (2.7) as an estimate of the error in the FC intracule from the 4389-term wave function and in the HF intracule from the 20-term wave function.

The calculated errors in the intracules using equation (2.6) and (2.7) are given in table 1. The errors in the helium and lithium data are of the order of 10^−9^. The anionic systems are not quite so accurate at this basis set size, but both the hydride ion and the critical nuclear charge systems have errors less than or equal to 2.4 × 10^−6^.

**Table 1.** The root-mean square error (RMS) and maximum error in the intracules.

	error in *D*_FC_(*r*)		error in *D*_HF_(*r*)	
system	RMS	max	RMS	max
Z_*C*_	2.333 × 10^−6^	1.489 × 10^−6^	1.235 × 10^−6^	6.366 × 10^−7^
H^−^	3.724 × 10^−8^	2.687 × 10^−8^	4.357 × 10^−7^	2.327 × 10^−7^
He	2.616 × 10^−9^	3.144 × 10^−9^	9.816 × 10^−9^	9.527 × 10^−9^
Li^+^	5.817 × 10^−9^	7.882 × 10^−9^	3.041 × 10^−9^	3.834 × 10^−9^

To determine the Coulomb hole curve Δ for a particular system, the FC and HF intracule distribution functions are subtracted point by point. In this work, 1000 data points between 0 and 20*a*_0_ for each intracule function were calculated. These data are provided in the electronic supplementary material in a downloadable format.

However, to determine the properties of the Coulomb hole, i.e. the root, area and minimum, the data points of Δ, i.e. Δ*D*(*r*) equation (2.5), were fitted to a spline curve, *f*(*a*), of the form:

2.8 $$f(a)=\;\{\begin{array}{ll} C_{0\text{,}0}+C_{1,0}a+C_{2,0}a^{2}+C_{3,0}a^{3} & a\text{ < }R_{0}\\ C_{0,1}+C_{1,1}a+C_{2,1}a^{2}+C_{3,1}a^{3} & a\text{ > }R_{1}\\ \begin{array}{cc} \;\vdots & \vdots \end{array} & \\ C_{0,n-1}+C_{1,n-1}a+C_{2,n-1}a^{2}+C_{3,n-1}a^{3} & a\text{ < }R_{n-1}\\ C_{0,n}+C_{1,n}a+C_{2,n}a^{2}+C_{3,n}a^{3} & \text{otherwise}, \end{array}$$

where *n* is the 1000 numerical data points between 0 and 20*a*_0_ used to form Δ from the *D*_FC_(*r*) and *D*_HF_(*r*) curves and the *R*_*i*_ values are the *r* values for each of these data points. The *C*_*j*,*i*_ are determined by the cubic spline fitting algorithm built into Maple, which was used to interpolate between the calculated data points of Δ giving improved estimates of the root, area and minimum of the Coulomb hole at reduced computational cost.

## 3. Results and discussion

### 3.1. Effects of electron correlation on some bound state properties

Table 2 provides the fully correlated, non-relativistic, three-body energy with infinite nuclear mass, *E*_FC_, and the Hartree–Fock energy, *E*_HF_, using the Laguerre-based methodologies described in the Material and method section. All energies are considered accurate to the number of digits provided; the errors in the energy are smaller than the errors in the intracule, table 1. Assuming *E*_*FC*_ ≈ *E*_exact_ then the correlation energy, *E*_corr_ is simply the difference; we have recently reported *E*_corr_ for (*Z* = *Z*_*C*_, 1, …, 18), [14]. The data demonstrate the importance of electron correlation, particularly for the anions (H^−^ and *Z*_*C*_) where HF predicts the system to have an energy greater than the lowest continuum threshold.

**Table 2.** Energy (a.u.), expectation values (a.u.), nucleus–electron cusp *ν*_31_ (exact value = −*Z*), electron–electron cusp *ν*_12_ (exact value = 0.5) and virial condition (exact value of *η* = 0), for *Z*_*C*_, H^−^, He and Li^+^ using either the fully correlated (FC)^a^ or the Hartree–Fock (HF)^b^ wave function. The correlation effect %Corr is calculated as (*X*_FC_ − *X*_HF_/*X*_FC_) × 100.

	*Z*_*C*_ = 0.911028224			H^−^		
property, *X*	FC^c^	HF	%Corr	FC	HF	%Corr
*E*	− 0.41498621	−0.37390622	9.899	−0.52775101654	−0.4879297343	7.545
〈*r*_1_〉	4.146	2.98900	27.906	2.710178278	2.503959	7.609
〈*r*_12_〉	7.083	4.4939	36.553	4.41269449	3.739273	15.260
〈*δ*(*r*_1_)〉	0.119094	0.1084	8.979	0.1645528	0.154	6.413
〈*δ*(*r*_12_)〉	0.001114	0.0086012	−672.100	0.002738	0.0129834763	−374.195
〈1/*r*_1_〉	0.578108	0.5957991	−3.060	0.68326176765	0.68567215	−0.352
〈1/*r*_12_〉	0.223374	0.337767	−51.211	0.3110215022	0.39548484	−27.156
〈*T̂*〉	0.41498621	0.186953113	54.949	0.5277510165	0.2439648671	53.772
〈*V̂*〉	−0.82997242	−0.37390622	54.949	−1.0555020330	−0.487929734	53.772
*ν*_31_	−0.9110	−0.9111	n.a.	−0.99999991	−1.00004	n.a.
*ν*_12_	0.4994	n.a.	n.a.	0.49998	n.a.	n.a.
*η*	2.49 × 10^−21^	2.20 × 10^−15^	n.a.	3.38 × 10^−20^	5.34 × 10^−14^	n.a.

	He			Li^+^		
	FC	HF	%Corr	FC	HF	%Corr
*E*	−2.90372437703	−2.861679995612	1.447	−7.27991341266	−7.2364152014522	0.597
〈*r*_1_〉	0.92947229487	0.9272734047	0.236	0.57277414997	0.572366815001	0.071
〈*r*_12_〉	1.42207025556	1.3621243836	4.215	0.8623153754	0.838314780311	2.783
〈*δ*(*r*_1_)〉	1.810429	1.797959	0.688	6.85200	6.83607	0.232
〈*δ*(*r*_12_)〉	0.106345	0.190603997806	−79.231	0.53372	0.770240340922	−44.314
〈1/*r*_1_〉	1.68831680071	1.687282215281	0.061	2.687924397	2.687419466644	0.018
〈1/*r*_12_〉	0.945818448	1.025768869	− 8.453	1.5677195591	1.651686396960	−5.355
〈*T̂*〉	2.9037243770	1.430839997806	50.723	7.279913412	3.61820760072612	50.298
〈*V̂*〉	−5.8074487540	−2.861679995612	50.723	−14.559826825	−7.2364152014522	50.298
*ν*_31_	−1.999996	−1.9999994	n.a.	−2.9999991	−2.9999992	n.a.
*ν*_12_	0.49995	n.a.	n.a.	0.499998	n.a.	n.a.
*η*	9.33 × 10^−22^	4.53 × 10^−17^	n.a.	3.96 × 10^−23^	7.12× 10^−19^	n.a.

^a^FC values reported agree with [19] for H^−^, [20] for He and [21] for Li^+^.

^b^For the HF values: 〈*r*_1_〉, 〈*r*^−1^_1_〉 and the cusps for helium agree with [22–24], respectively; the interparticle expectation values agree with the early work of [25] and the 〈*T*〉 and 〈*V*〉 values agree with [26].

^c^A 8436-term wave function was used for the reported cusp values and to establish the reported convergence of *Z*_*C*_ values at 4389.

To evaluate the effect of correlation on other key bound state properties, table 2 presents expectation values of the interparticle distances *r*_*i*_, including the two-particle Dirac delta functions, *δ*(*r*_*i*_), where

3.1 $$\langle \hat{X}\rangle =\langle \psi_{\mathrm{FC}}|\hat{X}|\psi_{\mathrm{FC}}\rangle \;\text{or}\;\langle \psi_{\mathrm{HF}}|\hat{X}|\psi_{\mathrm{HF}}\rangle .$$

Due to symmetry, the expectation values involving the nucleus–electron distance *r*_1_ are equal to those involving the nucleus–electron distance *r*_2_, so only the former are presented. Additionally, the quality of the wave functions can be evaluated by determining the extent to which the virial theorem and the two-particle cusp values are satisfied. For a Coulomb potential, the virial theorem takes the simple form, $2\langle \hat{T}\rangle =-\langle \hat{V}\rangle$. The expectation values of the potential and kinetic energy, $\left\langle \hat{V}\right\rangle$ and $\left\langle \hat{T}\right\rangle$, and $\eta =|\langle \hat{V}\rangle /\langle \hat{T}\rangle +2|$ are provided in table 2. The calculated values for *η* are less than 3.4 × 10^−20^ for the FC systems and 5.4 × 10^−14^ for the HF systems, close to the exact value of zero. The two-body cusp ratios are determined using [20,27]:

3.2 $$\nu_{ij}=\langle {\hat{\nu }}_{ij}\rangle =\frac{\langle \delta (r_{ij})(\partial /\partial r_{ij})\rangle }{\left\langle \delta (r_{ij})\right\rangle }.$$

The exact value of the nucleus–electron cusp *ν*_31_ is −*Z* for the infinite nuclear mass systems, and the exact value of the electron–electron cusp *ν*_12_ is 0.5 but is zero for Hartree–Fock systems. The Laguerre-based HF and FC wave functions provide a reasonable description of the nucleus–electron cusp *ν*_31_ for all systems. It is clear from the data presented that electron correlation has very little effect on *ν*_31_ and the effect (%Corr) diminishes with increasing *Z*.

In general, the data presented in table 2 are consistent with HF allowing the electrons to get too close as quantified by 〈*r*_12_〉; when the motion of the electrons is uncorrelated, the smaller inter-electronic separation results in a shorter average nucleus–electron distance, 〈*r*_1_〉. Thus, HF theory allows the electrons to get too close to the nucleus on average, in addition to each other.

To elucidate the electronic structure near the nucleus, the two-particle Dirac delta function 〈*δ*(*r*_1_ − *r*)〉 is calculated with *r* set to zero. The effect of electron correlation on the nucleus–electron (single-particle) probability density at the nucleus *ρ*(*r*) = 〈*δ*(*r*_1_)〉 is less than 1% for all but the anions, H^−^ and Z_*C*_, where the error introduced by the Hartree–Fock approximation is more pronounced. In each case, electron correlation serves to reduce the probability. However, the correlation effects are significantly larger for the intracule (electron-pair) density at *r*_12_ = 0 for all systems, and although the effect of correlation (%Corr) decreases with increasing nuclear charge as the nucleus–electron interaction begins to dominate [28], from 672% for *Z*_*C*_ to 44% for Li^+^, it is still significant. Consistent with HF allowing the electrons to get too close, electron correlation reduces the probability 〈*δ*(*r*_12_)〉.

Due to the subtle balance between nucleus–electron attraction and electron–electron repulsion, it is found that the average potential energy increases and thus the average kinetic energy decreases, by approximately 50% for all systems considered, thus maintaining the virial condition; although *η* is several orders of magnitude larger for the HF systems, *η* = 0 at the pico-scale for all systems considered (HF and FC).

A closer analysis of the potential energy terms, i.e. 〈1/*r*_1_〉 and 〈1/*r*_12_〉, demonstrates the significantly greater impact electron correlation has on all properties involving *r*_12_. In general, the effect of electron correlation %Corr decreases with increasing nuclear charge *Z* but the trend is independent of *Z*. The exception is for 〈1/*r*_1_〉 where for anions the electron correlation causes a slight increase whereas for *Z* = 2 or 3 the average value decreases slightly. This is the only property in table 2 where the anions have a slightly different behaviour to helium and the cation.

Overall, the virial condition, cusp condition and accurate expectation values, in addition to the good precision for the eigenvalues, indicate that a reasonable description for both the FC and HF eigenfunctions have been obtained. This provides us with confidence to explore the Coulomb holes.

### 3.2. Coulomb holes

**Figure 1.**
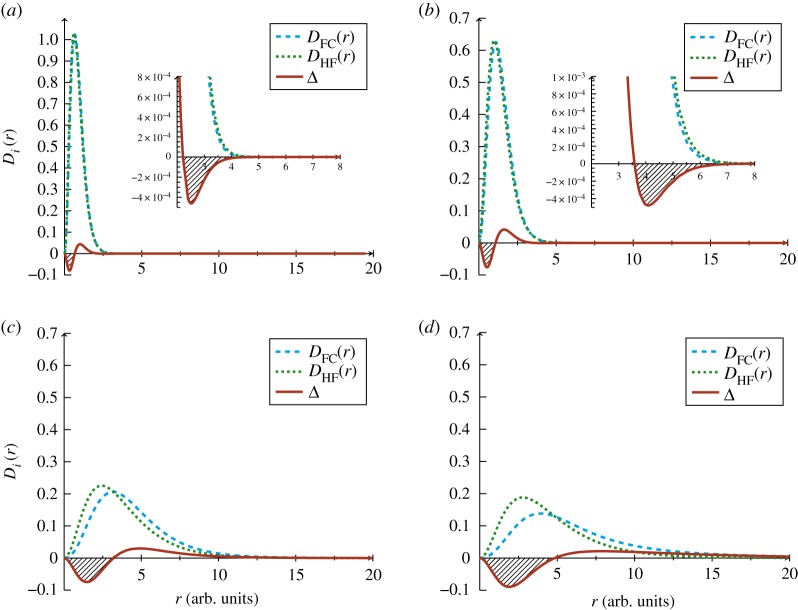
The Coulomb hole curve Δ (solid line), calculated as the difference between the intracule distribution function *D*_FC_(*r*) (dashed line) and *D*_HF_(*r*) (dotted line) for the singlet ground state of (*a*) Li^+^ (*b*) He (*c*) H^−^ and (*d*) *Z*_*C*_. The inset plot in (*a*,*b*) reveals the secondary Coulomb hole.

Figure 1 depicts the intracule distribution functions *D*_*i*_(*r*) where *i* =HF or FC, and their difference Δ*D*(*r*), equation (2.5), which gives rise to the Coulomb hole curve, which we refer to as Δ. The Coulomb holes are quantified and the key features of these holes (roots, area and minimum) are tabulated in table 3.

**Table 3.** Roots, areas and minima of the Coulomb holes in the helium-like ions in atomic units. Δ_1_ and Δ_2_ refer to the primary and secondary Coulomb holes, respectively.

system	$\Delta_{1}^{\min}$	$\Delta_{1}^{\mathrm{root}}$	$\Delta_{2}^{\min}$	$\Delta_{2}^{\mathrm{root}}$	$\Delta_{1}^{\mathrm{area}}$	$\Delta_{2}^{\mathrm{area}}$
*Z*_*C*_	1.91	4.85	—	—	2.30× 10^−1^	—
H^−^	1.46	3.20	—	—	1.33× 10^−1^	—
He	0.52	1.07	4.08	3.58	4.63× 10^−2^	6.12× 10^−4^
Li^+^	0.32	0.66	2.51	2.21	2.80× 10^−2^	3.50× 10^−4^

#### 3.2.1. Primary Coulomb holes

All systems considered: Li^+^, He, H^−^ and the critical nuclear charge system *Z*_*C*_ exhibit a Coulomb hole at short inter-electronic separation, shaded area between zero and the first root in figure 1*a*,*b*. It is well understood that in the HF mean-field approach to electron–electron repulsion, the electrons are allowed to get too close together, as quantified in table 2. The Coulomb hole represents an area around each electron where another electron is unlikely to be found, as the effect of electron correlation, the instantaneous interaction between electrons, is for the electrons to move to greater separation due to charge repulsion. The Coulomb hole is negative for small *r* and positive for larger *r* as a consequence of correlation shifting the FC intracule away from the origin relative to the HF intracule such that the HF system always has a greater probability at these radial separations.

The effect of electron correlation is more pronounced for anionic systems demonstrated by the much greater difference in the features of the diffuse HF and FC intracules, figure 1*c*,*d*. The most probable separation (*r*_max_) for H^−^ increases from 2.43*a*_0_ in the mean-field approach of HF to 3.17*a*_0_ as a result of electron correlation and the corresponding probability at these values is reduced by over 8%. This is amplified for *Z*_*C*_ where $r_{\max}$ shifts from 2.77*a*_0_ in the mean-field approach of HF to 3.92*a*_0_ as a result of electron correlation, and the probability at *r*_max_ is decreased by over 26% in the correlated system. This is to be compared with the values for helium, where electron correlation increases the most probable separation by approximately 10% but the probability density at *r*_max_ changes by less than 1%.

The radius of the Coulomb hole for H^−^ is three times greater than for helium (50% greater when *r* is *Z*-scaled), and for *Z*_*C*_ it is even greater. The Coulomb hole curve for H^−^ shows that the probability of the two electrons lying anywhere within a distance of 3.2*a*_0_ from each other is less than it would be without correlation (HF), and correspondingly the probability that the electrons are separated by more than 3.2*a*_0_ is greater. Furthermore, although the net content of the hole is zero as each intracule is normalized, the total amount of charge displaced by the Coulomb hole is equal to the area of the curve between zero and root 1, labelled $\Delta_{1}^{\min}$ in table 3 [11]. For H^−^, this is the area between *r*_12_ = 0 and *r*_12_ = 3.20*a*_0_ which corresponds to 0.133e. This is to be compared with the value for helium which is approximately one-twentieth of an electron. Perhaps surprisingly, just prior to electron detachment at *Z*_*C*_, the charge displaced is still just approximately one-fifth of an electron. Furthermore, it is worth noting that even at *Z*_*C*_, the detaching electron remains localized at a finite distance from the nucleus [14–16]. An explanation for this behaviour, presented in [16], is that the system transitions from a bound state to a shape resonance as the nuclear charge goes through the critical point. Mathematically, it was proven that the bound state does not spread as it approaches the dissociation threshold [29,30].

**Figure 2.**
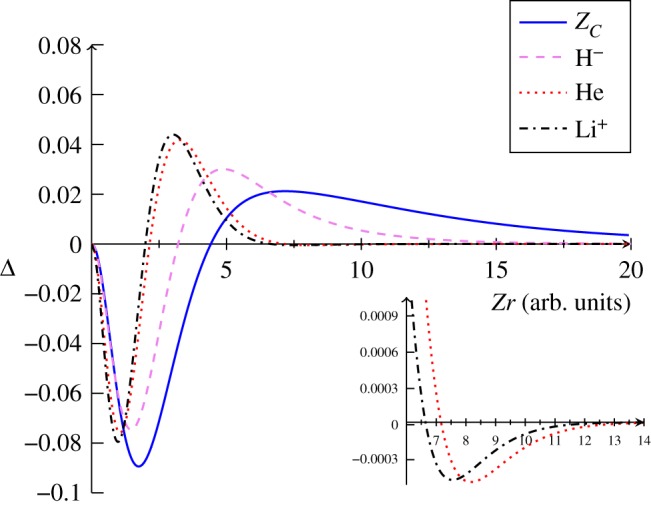
The *Z*-scaled Coulomb hole curves Δ for the ground state helium-like ions.

To provide a direct comparison of the Coulomb holes for the four systems, the *Z*-scaled intracule distributions are shown in figure 2. The Coulomb holes for the anions exhibit a greater radius and displace a greater charge and even at 10*a*_0_, which is over 5 Å, the probability is still significant, contrary to the Coulomb holes for helium and the lithium cation which contract toward the origin with increasing *Z* as the nucleus–electron attraction competes successfully with the electron–electron repulsion [28].

#### 3.2.2. Secondary Coulomb holes and long-range behaviour

In agreement with the conclusions of Pearson *et al.* [13], we confirm the existence of a secondary hole for He and Li^+^ which corresponds to a decrease in the probability of finding the electrons far apart, see inset in figure 1*a*,*b*. This results from the intracule distribution functions having a second crossing point indicating that, although at shorter distances (*r*_12_ < 3.58*a*_0_ ≈ 1.89 Å for He and *r*_12_ < 2.21*a*_0_ ≈ 1.17 Å for Li^+^) the mean-field approach of HF allows electrons to get too close, at larger separations the electron correlation decreases the probability of two electrons being apart. The key features of both the primary Coulomb hole and the secondary Coulomb hole for the helium atom and the lithium cation are in good agreement with those reported by Pearson *et al.* [13]. A second Coulomb hole has also been observed for H_2_ [31].

**Figure 3.**
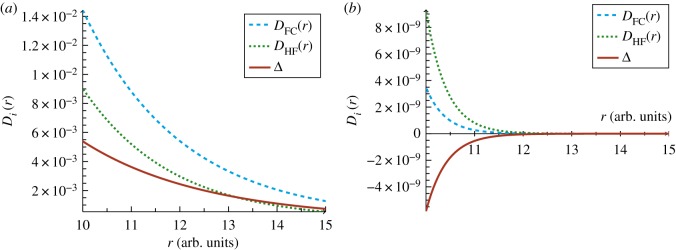
Long-range behaviour of the intracule distribution functions (dashed and dotted lines) and their difference giving rise to the Coulomb hole curve (solid line) for (*a*) H^−^ and (*b*) He.

Particularly interesting is the long-range behaviour of the electron-pair density with and without electron correlation for H^−^ and *Z*_*C*_. In addition to the most probable distance shifting to a much greater value, electron correlation effects result in non-zero probability over a much wider range of separations. There is no evidence of a secondary Coulomb hole for either H^−^ or the *Z*_*C*_ system, and at all distances, electron correlation increases the probability of the electrons being apart. In fact, even at 10*a*_0_, the probability density is still greater than 10^−3^ for H^−^, figure 3*a*, contrary to helium where the probability density is of the order of 10^−9^ at these radial separations, figure 3*b*. Furthermore, electron correlation becomes increasingly important at larger separations. Beyond about 13.5*a*_0_ the difference (Coulomb hole curve, solid line) becomes larger than the HF intracule (dotted line) figure 3*a*, and as the probability density approaches the value for the second Coulomb hole in helium (i.e. ≈10^−4^), the Coulomb hole curve starts to align with the characteristics of the correlated intracule. This indicates that the statistically independent and uncorrelated motion of the electrons in the HF approach, which increases the likelihood of finding the electrons close together at short distances, decreases the probability of their being far apart at large separations more rapidly than for electrons in which their motion is correlated.

#### 3.2.3. Tertiary Coulomb holes?

It has been suggested that additional holes may exist at even larger values of *r*_12_ [13]; however, at the level of accuracy used in the present study, we found no evidence of additional Coulomb holes, figure 3*b*. This also indicates that the counterintuitive conclusion for heliogenic systems with *Z* ≥ 2, that electron correlation increases the probability of finding the electrons at these distances, is true at all distances greater than the root of the secondary hole $\Delta_{2}^{\mathrm{root}}$, table 3.

#### 3.2.4. Emergence of secondary Coulomb holes

The conclusion of Pearson *et al.* [13] that electron correlation can act to bring distant electrons closer together is counterintuitive. We confirm their findings using densities at least three orders of magnitude more accurate and extend their analysis to show that H^−^ does not appear to have a secondary Coulomb hole. This gives rise to two key questions: (1) How can a Coulomb interaction bring two like-charged particles closer together? and (2) At what nuclear charge value, does a secondary Coulomb hole emerge?

We attribute the formation of the secondary Coulomb hole to the subtle interplay between the nucleus–electron attraction and electron–electron repulsion. The secondary Coulomb hole provides information about the long-range behaviour of the electron–electron interaction. As *Z* increases, the electrons are drawn closer to the nucleus. Previously, we showed that the inner electron in a two-electron system experiences a negative screening effect due to the perturbation by the other electron; this effect increases with increasing *Z*, and the balance of the nucleus–electron attraction and the electron–electron repulsion determines the magnitude of |〈*r*_in_〉 − 〈*r*_out_〉|, [28]. In this work, as *Z* increases and the electrons are drawn closer to the nucleus, the correlated electrons can adjust their relative positions and maximize the attractive interaction. Thus, for *Z* ≥ 2, the probability that the two electrons will be found at large separations reduces more rapidly than when the electronic motions are uncorrelated, i.e. *D*_FC_(*r*) → 0 faster than *D*_HF_(*r*) → 0. The FC and HF intracule curves cross, resulting in a second Coulomb hole at larger separations.

In the case of the anions, the nuclear charge is not sufficient to significantly overcome the electron–electron repulsion and so the curves do not cross a second time. At all distances, electron correlation increases the probability of the electrons being apart and at very large separations the Coulomb hole curve aligns with the FC intracule as the HF intracule goes to zero, i.e. *D*_HF_(*r*) → 0 faster than *D*_FC_(*r*) → 0. This non-zero probability at large *r*_12_, due to electron correlation, is responsible for H^−^ containing a bound state when HF predicts H^−^ to be unbound (i.e. HF energy for H^−^ is higher than the ground state energy of a hydrogen atom).

**Figure 4.**
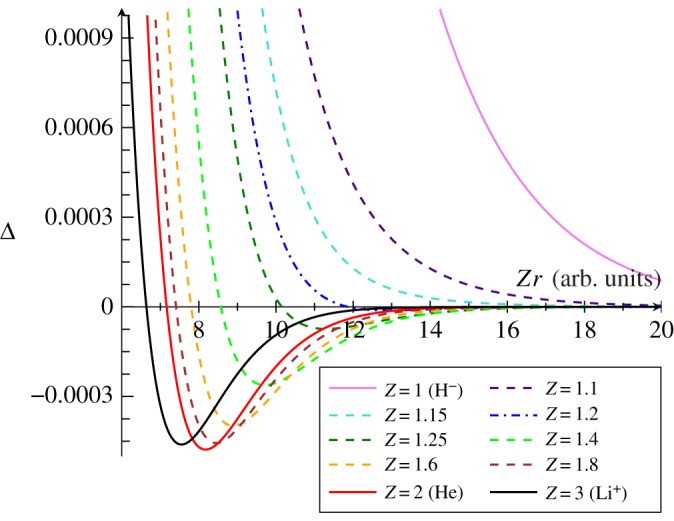
The *Z*-scaled second Coulomb hole curves Δ, for arbitrary charge, *Z* systems.

To determine the nuclear charge required for a secondary Coulomb hole to emerge, the HF and FC intracules for non-integer nuclear charge values between *Z* = 1 corresponding to the hydride ion and *Z* = 2 corresponding to helium were calculated, in increments of 0.1, and used to determine Coulomb hole data. The *Z*-scaled Coulomb hole curves are presented in figure 4 and quantified in table 4. The data indicate that a secondary Coulomb hole for a heliogenic system emerges when *Z* = 1.2; it has an area of 2.73 × 10^−5^ which is ≈12 times smaller than the area of the secondary Coulomb hole for the system with *Z* = 1.3.

**Table 4.** Roots, areas and minima of the Coulomb holes for heliogenic systems with non-integer nuclear charge *Z* in atomic units. Δ_1_ and Δ_2_ refer to the primary and secondary Coulomb holes, respectively.

system	$\Delta_{1}^{\min}$	$\Delta_{1}^{\mathrm{root}}$	$\Delta_{2}^{\min}$	$\Delta_{2}^{\mathrm{root}}$	$\Delta_{1}^{\mathrm{area}}$	$\Delta_{2}^{\mathrm{area}}$
H^−^	1.46	3.20	—	—	1.33× 10^−1^	—
*Z* = 1.1	1.22	2.57	—	—	1.04× 10^−1^	—
*Z* = 1.15	1.13	2.37	—	—	9.54× 10^−2^	—
*Z* = 1.2	1.05	2.20	10.78	9.89	8.86× 10^−2^	2.73× 10^−5^
*Z* = 1.25	0.99	2.05	9.04	8.11	8.83× 10^−2^	1.79× 10^−4^
*Z* = 1.3	0.93	1.93	8.11	7.22	7.83× 10^−2^	3.36× 10^−4^
*Z* = 1.4	0.84	1.73	6.93	6.12	7.07× 10^−2^	5.53× 10^−4^
*Z* = 1.5	0.76	1.56	6.14	5.40	6.47× 10^−2^	6.57× 10^−4^
*Z* = 1.6	0.70	1.43	5.55	4.87	5.97× 10^−2^	6.94× 10^−4^
*Z* = 1.7	0.64	1.32	5.08	4.45	5.56× 10^−2^	6.93× 10^−4^
*Z* = 1.8	0.60	1.22	4.69	4.11	5.20× 10^−2^	6.73× 10^−4^
*Z* = 1.9	0.56	1.01	4.36	3.82	4.89× 10^−2^	6.45× 10^−4^
He	0.52	1.07	4.08	3.58	4.63× 10^−2^	6.12× 10^−4^

## 4. Conclusion

We have calculated the Coulomb hole(s) in the lithium cation, helium atom, hydride ion, and the system with the critical nuclear charge for binding two electrons to model the long-range, low-density behaviour of the intracule density. To maximize accuracy, and to provide a balanced description of the Coulomb holes, both the reference HF method and the fully correlated method are implemented using a Laguerre-based wave function with a single nonlinear parameter. An additional advantage of these implementations is that, for the most part, it can be solved by series solution using the Laguerre recursion relations which results in good computational speed, and both methods model the electron–nucleus cusp behaviour correctly as shown in table 2.

The errors in the intracule for helium and the lithium cation are of the order of 10^−9^, much smaller than in previous work. The Coulomb holes for these systems were identified and characterized. It was found, as in previous work [13], that they exhibit a primary hole at small separations and a secondary hole at larger radial separation. The charge at which a secondary Coulomb hole emerges was investigated and found to occur between *Z* = 1.15 and *Z* = 1.20. Higher Coulomb holes were sought but no evidence of additional Coulomb holes was found at the current level of accuracy.

High-accuracy data have been presented for the H^−^ ion and the system with a critical nuclear charge for binding two electrons, *Z*_*C*_. The known inadequacies of HF are further exemplified by the characterization of the Coulomb hole for these anions. It is shown that the effect of electron correlation is to shift the most probable electron–electron distance *r*_max_ to larger separations and the probability at *r*_max_ decreases as the distribution becomes more diffuse. The uncorrelated electronic motion described by the HF intracule does not capture the long-range behaviour of a correlated system and thus the FC intracule density dominates the Coulomb hole behaviour at larger separations. This is a manifestation of the HF prediction that the hydride ion is unstable to electron detachment.

In summary, it is hoped that this accurate characterization of intracule densities and Coulomb hole characteristics at long range and low density will serve as a stringent and useful test to benchmark new method developments that seek to extend the domain of reliability and chemical accuracy to more complex and exotic chemical regimes.

## Supplementary Material

Coulomb hole supplementary

## Data accessibility

A complete dataset of intracule and Coulomb hole data for each system (i.e. *Z*_*C*_, H^−^, He and Li^+^) is available from the Dryad Digital Repository (http://dx.doi.org/10.5061/dryad.r60sj21) [32].
