# Supplementary material for: Electron correlation in Li+, He, H− and the critical nuclear charge system ZC: energies, densities and Coulomb holes
Source: R Soc Open Sci. 2019 Jan 9;6(1):181357. doi: 10.1098/rsos.181357 (PMC6366201; doi:10.1098/rsos.181357)
Supplement: Coulomb hole supplementary [file rsos181357supp1.pdf]

SUPPLEMENTARY MATERIAL

Electron correlation in Li^+, He, H^- and the critical nuclear charge system Z_C: energies, densities and Coulomb holes

Adam L. Baskerville, Andrew W. King[a] and Hazel Cox[†]

Department of Chemistry, School of Life Sciences,

University of Sussex, Falmer, Brighton BN1 9QJ, U.K.

[a] Present Address: Department of Biochemistry, Faculty of Science, Chulalongkorn University, Bangkok 10330, Thailand.

[†] h.cox@sussex.ac.uk

CONTENTS

I. Fully Correlated (FC) data	3
A. The FC Wavefunctions	3
B. FC energy convergence data	5
1. One non-linear variational parameter (α)	5
2. Two non-linear variational parameters (α, γ)	6
C. FC expectation value convergence data	7
1. One non-linear variational parameter (α)	7
2. Two non-linear variational parameters (α, γ)	11
II. Hartree Fock (HF) data	15
III. Intracule and Coulomb hole data	15
References	15

I. FULLY CORRELATED (FC) DATA

A. The FC Wavefunctions

The following is the form of the FC wavefunction in perimetric coordinates (z_i):

$$\Psi(z_1, z_2, z_3) = e^{-\frac{1}{2}(\alpha z_1+\beta z_2+\gamma z_3)} \sum_{l,m,n=0}^{\infty} A(l,m,n)L_l(\alpha z_1)L_m(\beta z_2)L_n(\gamma z_3) \tag{1}$$

and in inter-particle coordinates (r_i):

$$\Psi(r_1, r_2, r_3) = e^{-(Ar_1+Br_2+Cr_3)} \sum_{l,m,n=0}^{\infty} A(l,m,n)L_l(Ar_1)L_m(Br_2)L_n(Cr_3). \tag{2}$$

The relations to convert between α, γ and A, C are as follows:

$$\begin{aligned} \alpha &= B + C, \quad A = \frac{\beta+\gamma-\alpha}{2} \\ \beta &= A + C, \quad B = \frac{\alpha+\gamma-\beta}{2} \\ \gamma &= A + B, \quad C = \frac{\alpha+\beta-\gamma}{2}. \end{aligned} \tag{3}$$

When $\alpha = \beta$, α and γ are varied independently and this is referred to as the two-parameter (α, γ) wavefunction. It corresponds to non-zero values of $A = B$ and C in the exponent of equation (2).

When $\alpha = \beta$, and $\gamma = 2\alpha$ the exponent in the wave function (equation (1)) models, in principle, the correct asymptotic behaviour of the solution of the Schrödinger equation for two-electron atoms at large r_1 and r_2 as using the relations in equation (3) results in $A = B$ and $C = 0$ in equation (2). Here, only one parameter, α is varied. This is referred to as the one-parameter wavefunction and is the wavefunction used for the data reported in the main paper.

Data (energies and expectation values) from both the one-parameter and two-parameter wavefunctions are reported here in the SM for comparison purposes. The optimised non-linear variational parameters for a 4389-term wavefunction for each system is provided in Table I and Table II.

TABLE I: The optimised value of α in the one-parameter (α) perimetric wavefunction (and the resulting value of A, see equation (2)) for the 4389-term wavefunction used to generate the data provided in the main paper.

System	α	A
Z_C	0.43	0.43
H^-	1.22	1.22
He	2.74	2.74
Li^+	3.10	3.10

TABLE II: The optimised values of α and γ in the two-parameter (α, γ) perimetric wavefunction (and the resulting values of A and C, see equation (2)) for 4389-term wavefunctions.

System	α	γ	A	C
Z_C	0.43	1.44	0.72	-0.29
H^-	1.23	2.49	1.24	-0.01
He	4.82	8.19	4.09	1.45
Li^+	4.58	6.48	3.24	1.34

B. FC energy convergence data

1. One non-linear variational parameter (α)

TABLE III: Convergence of the ground state energy with increasing basis set size for the Fully correlated (FC) implementation with one non-linear variational parameter (α). Electronic energies in a.u.

No. Terms	Z_C	H^-	He	Li^+
22	-0.414 465 215 007	-0.527 669 735 063	-2.903 713 945 024	-7.279 902 014 123
95	-0.414 955 459 163	-0.527 750 064 292	-2.903 724 305 388	-7.279 913 342 574
161	-0.414 977 181 421	-0.527 750 865 613	-2.903 724 366 434	-7.279 913 402 674
252	-0.414 983 240 805	-0.527 750 985 998	-2.903 724 375 039	-7.279 913 410 840
444	-0.414 985 530 222	-0.527 751 012 070	-2.903 724 376 769	-7.279 913 412 451
1078	-0.414 986 157 473	-0.527 751 016 377	-2.903 724 377 026	-7.279 913 412 663
2856	-0.414 986 209 710	-0.527 751 016 541	-2.903 724 377 031	-7.279 913 412 665
4389	-0.414 986 211 724	-0.527 751 016 543	-2.903 724 377 032	-7.279 913 412 666

2. *Two non-linear variational parameters (α, γ)*

TABLE IV: Convergence of the ground state energy with increasing basis set size for the Fully correlated (FC) implementation with two non-linear variational parameters (α, γ). Electronic energies in a.u.

No. Terms	Z_C	H^-	He	Li^+
22	-0.414 546 549 473	-0.527 679 151 441	-2.903 715 033 193	-7.279 903 229 470
95	-0.414 959 635 965	-0.527 750 090 417	-2.903 724 312 074	-7.279 913 347 994
161	-0.414 978 195 616	-0.527 750 868 164	-2.903 724 367 391	-7.279 913 403 418
252	-0.414 983 517 155	-0.527 750 986 302	-2.903 724 375 214	-7.279 913 412 472
444	-0.414 985 580 772	-0.527 751 012 081	-2.903 724 376 779	-7.279 913 412 473
1078	-0.414 986 161 596	-0.527 751 016 377	-2.903 724 377 026	-7.279 913 412 661
2856	-0.414 986 209 496	-0.527 751 016 540	-2.903 724 377 034	-7.279 913 412 665
4389	-0.414 986 211 780	-0.527 751 016 543	-2.903 724 377 034	-7.279 913 412 666

C. FC expectation value convergence data

1. *One non-linear variational parameter (α)*

TABLE V: Values of various expectation values for the critical nuclear charge system (Z_C) with increasing basis set size for the Fully Correlated (FC) implementation. The critical nuclear charge value is $Z_C = 0.911\ 028\ 224\ 077\ 255\ 73(4)$. A 8436-term wavefunction was used to establish convergence at 4389.

No. terms	$\langle r_1 \rangle$	$\langle r_{12} \rangle$	$\left\langle \frac{1}{r_1} \right\rangle$	$\left\langle \frac{1}{r_{12}} \right\rangle$	$\langle \delta(r_1) \rangle$
22	3.501 514 982 617	5.828 316 961 362	0.587 198 426 155	0.240 978 247 955	0.116 268 593 744
95	3.964 662 191 378	6.724 845 249 670	0.579 690 850 226	0.226 318 532 451	0.118 601 486 928
161	4.055 324 964 412	6.902 602 358 918	0.578 763 124 226	0.224 584 719 581	0.118 880 244 297
252	4.100 545 939 589	6.991 627 681 885	0.578 390 007 933	0.223 892 762 105	0.118 997 614 208
444	4.129 039 306 854	7.047 893 609 605	0.578 198 146 959	0.223 538 601 407	0.119 055 720 159
1078	4.144 019 322 435	7.077 560 921 712	0.578 119 401 809	0.223 393 868 883	0.119 086 987 482
2856	4.146 643 031 232	7.082 772 000 239	0.578 109 515 593	0.223 375 751 640	0.119 093 950 202
4389	4.146 852 863 957	7.083 189 473 686	0.578 108 914 963	0.223 374 652 810	0.119 094 453 703
8436	4.146 945 027 272	7.083 372 972 803	0.578 108 687 501	0.223 374 236 947	0.119 094 912 843
	$\langle \delta(r_{12}) \rangle$	$\langle \hat{T} \rangle$	$\langle \hat{V} \rangle$	ν_{21}	ν_{31}
22	0.001 339 968 408	0.414 465 215 759	-0.828 930 430 766	0.411 782 141 579	-0.889 196 462 499
95	0.001 165 440 395	0.414 955 459 974	-0.829 910 919 138	0.457 465 117 389	-0.905 122 390 415
161	0.001 142 403 471	0.414 977 181 447	-0.829 954 362 869	0.468 912 126 646	-0.907 925 610 416
252	0.001 130 573 193	0.414 983 240 592	-0.829 966 481 397	0.477 445 534 658	-0.909 344 890 978
444	0.001 122 003 988	0.414 985 530 347	-0.829 971 060 570	0.486 113 870 932	-0.910 190 296 020
1078	0.001 116 704 635	0.414 986 157 512	-0.829 972 314 986	0.493 915 026 439	-0.910 770 622 931
2856	0.001 115 013 422	0.414 986 209 488	-0.829 972 418 985	0.497 801 501 336	-0.910 986 582 103
4389	0.001 114 766 045	0.414 986 211 709	-0.829 972 423 434	0.498 752 168 222	-0.910 993 193 382
8436	0.001 114 602 981	0.414 986 212 421	-0.829 972 424 848	0.499 455 312 510	-0.911 018 410 363

TABLE VI: Values of various expectation values for the hydride anion (H^-) with increasing basis set size for the Fully Correlated (FC) implementation.

No. terms	$\langle r_1 \rangle$	$\langle r_{12} \rangle$	$\left\langle \frac{1}{r_1} \right\rangle$	$\left\langle \frac{1}{r_{12}} \right\rangle$	$\langle \delta(r_1) \rangle$
22	2.685 429 488 615	4.365 204 068 253	0.683 808 212 279	0.312 276 952 761	0.163 358 393 804
95	2.709 585 725 994	4.411 541 174 270	0.683 270 563 281	0.311 040 998 237	0.164 527 369 265
161	2.710 066 349 413	4.412 475 863 973	0.683 263 221 353	0.311 024 711 279	0.164 549 340 374
252	2.710 152 837 488	4.412 644 685 464	0.683 262 066 008	0.311 022 159 970	0.164 552 780 071
444	2.710 174 383 215	4.412 686 851 929	0.683 261 807 610	0.311 021 591 069	0.164 548 381 117
1078	2.710 178 103 505	4.412 694 153 737	0.683 261 769 210	0.311 021 505 684	0.164 552 283 116
2856	2.710 178 274 878	4.412 694 490 955	0.683 261 767 674	0.311 021 502 273	0.164 552 870 932
4389	2.710 178 278 063	4.412 694 497 236	0.683 261 767 651	0.311 021 502 219	0.164 552 856 533
	$\langle \delta(r_{12}) \rangle$	$\left\langle \hat{T} \right\rangle$	$\left\langle \hat{V} \right\rangle$	ν_{21}	ν_{31}
22	0.002 891 525 980	0.527 669 736 733	-1.055 339 471 797	0.430 250 527 555	-0.989 852 341 315
95	0.002 756 438 350	0.527 750 064 033	-1.055 500 128 326	0.483 222 868 458	-0.999 450 907 335
161	0.002 745 568 879	0.527 750 865 812	-1.055 501 731 426	0.491 073 350 287	-0.999 865 951 740
252	0.002 741 405 576	0.527 750 986 046	-1.055 501 972 045	0.494 986 120 974	-0.999 972 434 665
444	0.002 739 128 885	0.527 751 012 081	-1.055 502 024 151	0.497 813 771 319	-0.999 827 123 009
1078	0.002 738 182 390	0.527 751 016 358	-1.055 502 032 736	0.499 424 574 170	-0.999 963 594 430
2856	0.002 738 015 485	0.527 751 016 534	-1.055 502 033 075	0.499 883 602 609	-0.999 999 671 378
4389	0.002 738 001 933	0.527 751 016 539	-1.055 502 033 083	0.499 981 038 844	-0.999 999 911 349

TABLE VII: Values of various expectation values for helium (He) with increasing basis set size for the Fully Correlated (FC) implementation.

No. terms	$\langle r_1 \rangle$	$\langle r_{12} \rangle$	$\left\langle \frac{1}{r_1} \right\rangle$	$\left\langle \frac{1}{r_{12}} \right\rangle$	$\langle \delta(r_1) \rangle$
22	0.929 421 479 388	1.421 972 811 828	1.688 316 800 404	0.945 839 311 557	1.811 066 704 502
95	0.929 471 853 486	1.422 069 424 010	1.688 316 806 262	0.945 818 614 294	1.810 422 039 156
161	0.929 472 224 038	1.422 070 122 137	1.688 316 801 839	0.945 818 474 545	1.810 422 746 583
252	0.929 472 280 603	1.422 070 228 660	1.688 316 800 969	0.945 818 453 830	1.810 425 715 552
444	0.929 472 292 482	1.422 070 251 046	1.688 316 800 785	0.945 818 449 572	1.810 425 818 935
1078	0.929 472 294 838	1.422 070 255 498	1.688 316 800 707	0.945 818 448 813	1.810 428 928 552
2856	0.929 472 294 875	1.422 070 255 568	1.688 316 800 711	0.945 818 448 800	1.810 429 159 790
4389	0.929 472 294 874	1.422 070 255 567	1.688 316 800 713	0.945 818 448 800	1.810 429 192 375
	$\langle \delta(r_{12}) \rangle$	$\left\langle \hat{T} \right\rangle$	$\left\langle \hat{V} \right\rangle$	ν_{21}	ν_{31}
22	0.107 161 912 084	2.903 713 945 034	-5.807 427 890 059	0.468 387 724 607	-2.002 005 190 500
95	0.106 404 645 618	2.903 724 305 364	-5.807 448 610 753	0.495 159 659 263	-1.999 963 757 193
161	0.106 366 147 310	2.903 724 366 378	-5.807 448 732 812	0.497 768 138 525	-1.999 949 742 843
252	0.106 353 527 233	2.903 724 375 008	-5.807 448 750 047	0.498 892 179 982	-1.999 964 384 100
444	0.106 347 719 195	2.903 724 376 799	-5.807 448 753 568	0.499 560 884 102	-1.999 952 206 731
1078	0.106 345 704 645	2.903 724 376 992	-5.807 448 754 018	0.499 902 984 368	-1.999 991 655 146
2856	0.106 345 530 829	2.903 724 377 015	-5.807 448 754 046	0.499 945 420 728	-1.999 996 013 451
4389	0.106 345 497 056	2.903 724 377 020	-5.807 448 754 051	0.499 954 620 132	-1.999 996 666 150

TABLE VIII: Values of various expectation values for the lithium ion (Li^+) with increasing basis set size for the Fully Correlated (FC) implementation.

No. terms	$\langle r_1 \rangle$	$\langle r_{12} \rangle$	$\left\langle \frac{1}{r_1} \right\rangle$	$\left\langle \frac{1}{r_{12}} \right\rangle$	$\langle \delta(r_1) \rangle$
22	0.572 763 605 243	0.862 294 837 104	2.687 923 776 210	1.567 738 631 097	6.853 005 336 927
95	0.572 774 064 622	0.862 315 217 027	2.687 924 396 139	1.567 719 691 660	6.851 949 457 007
161	0.572 774 136 933	0.862 315 351 352	2.687 924 397 326	1.567 719 578 690	6.851 981 004 156
252	0.572 774 147 417	0.862 315 370 742	2.687 924 397 438	1.567 719 562 824	6.851 996 688 812
444	0.572 774 149 619	0.862 315 374 818	2.687 924 397 396	1.567 719 559 600	6.852 002 137 874
1078	0.572 774 149 967	0.862 315 375 448	2.687 924 397 406	1.567 719 559 147	6.852 008 580 484
2856	0.572 774 149 971	0.862 315 375 456	2.687 924 397 406	1.567 719 559 141	6.852 008 848 301
4389	0.572 774 149 975	0.862 315 375 462	2.687 924 397 381	1.567 719 559 159	6.852 007 719 354
	$\langle \delta(r_{12}) \rangle$	$\left\langle \hat{T} \right\rangle$	$\left\langle \hat{V} \right\rangle$	ν_{21}	ν_{31}
22	0.536 093 002 076	7.279 902 012 041	-14.559 804 026 165	0.471 049 306 277	-3.001 671 507 109
95	0.533 884 760 077	7.279 913 342 599	-14.559 826 685 173	0.495 741 145 376	-2.999 864 049 109
161	0.533 778 131 438	7.279 913 402 592	-14.559 826 805 267	0.498 073 788 906	-2.999 908 392 299
252	0.533 743 915 817	7.279 913 410 966	-14.559 826 821 807	0.499 061 357 964	-2.999 946 733 490
444	0.533 728 543 268	7.279 913 412 326	-14.559 826 824 778	0.499 635 732 517	-2.999 957 131 980
1078	0.533 723 361 433	7.279 913 412 630	-14.559 826 825 292	0.499 922 166 533	-2.999 992 142 294
2856	0.533 723 152 136	7.279 913 412 634	-14.559 826 825 298	0.499 938 230 174	-2.999 994 276 760
4389	0.533 724 245 478	7.279 913 412 485	-14.559 826 825 132	0.499 998 810 998	-2.999 999 123 943

2. Two non-linear variational parameters (α, γ)

TABLE IX: Values of various expectation values for the critical nuclear charge system (Z_C) with increasing basis set size for the Fully Correlated (FC) implementation. The critical nuclear charge value is $Z_C = 0.911\ 028\ 224\ 077\ 255\ 73(4)$. A 8436-term wavefunction using 1 parameter (α) was used to establish convergence at 4389.

No. terms	$\langle r_1 \rangle$	$\langle r_{12} \rangle$	$\left\langle \frac{1}{r_1} \right\rangle$	$\left\langle \frac{1}{r_{12}} \right\rangle$	$\langle \delta(r_1) \rangle$
22	3.550 216 354 763	5.922 522 164 609	0.586 168 158 720	0.238 938 374 216	0.116 900 234 512
95	3.982 553 206 435	6.759 841 558 600	0.579 482 575 075	0.225 930 690 786	0.118 699 687 149
161	4.063 883 136 290	6.919 421 261 265	0.578 683 985 024	0.224 438 495 065	0.118 919 947 414
252	4.104 354 122 758	6.999 136 559 581	0.578 361 151 905	0.223 839 631 884	0.119 015 288 819
444	4.130 090 754 130	7.049 973 862 322	0.578 191 956 678	0.223 527 221 399	0.119 054 245 894
1078	4.144 213 568 127	7.077 946 437 724	0.578 118 585 625	0.223 392 373 513	0.119 086 142 213
2856	4.146 664 238 888	7.082 814 182 785	0.578 109 451 445	0.223 375 634 316	0.119 094 285 380
4389	4.146 860 246 209	7.083 204 167 467	0.578 108 895 430	0.223 374 617 098	0.119 094 297 984
8436	4.146 945 027 272	7.083 372 972 803	0.578 108 687 501	0.223 374 236 947	0.119 094 912 843
	$\langle \delta(r_{12}) \rangle$	$\langle \hat{T} \rangle$	$\langle \hat{V} \rangle$	ν_{21}	ν_{31}
22	0.001 338 729 540	0.414 546 562 386	-0.829 093 125 355	0.404 245 350 346	-0.894 968 056 911
95	0.001 166 997 514	0.414 959 635 757	-0.829 919 271 722	0.453 553 752 070	-0.906 227 205 763
161	0.001 143 385 057	0.414 978 195 676	-0.829 956 391 292	0.466 708 231 101	-0.908 395 519 030
252	0.001 131 066 641	0.414 983 517 151	-0.829 967 034 307	0.476 249 858 293	-0.909 564 696 107
444	0.001 122 114 552	0.414 985 580 764	-0.829 971 161 536	0.485 685 279 457	-0.910 120 207 904
1078	0.001 116 759 998	0.414 986 161 626	-0.829 972 323 222	0.493 680 701 050	-0.910 730 200 164
2856	0.001 115 032 928	0.414 986 209 718	-0.829 972 419 428	0.497 802 205 260	-0.910 873 250 918
4389	0.001 114 776 733	0.414 986 211 775	-0.829 972 423 556	0.498 685 124 281	-0.910 981 857 536
8436	0.001 114 602 981	0.414 986 212 421	-0.829 972 424 848	0.499 455 312 511	-0.911 018 410 363

TABLE X: Values of various expectation values for the hydride anion (H^-) with increasing basis set size for the Fully Correlated (FC) implementation.

No. terms	$\langle r_1 \rangle$	$\langle r_{12} \rangle$	$\left\langle \frac{1}{r_1} \right\rangle$	$\left\langle \frac{1}{r_{12}} \right\rangle$	$\langle \delta(r_1) \rangle$
22	2.689 284 956 482	4.372 592 822 802	0.683 697 220 910	0.312 036 138 923	0.163 651 026 427
95	2.709 613 490 923	4.411 595 714 298	0.683 270 046 326	0.311 039 911 828	0.164 543 191 725
161	2.710 070 133 324	4.412 483 332 963	0.683 263 159 182	0.311 024 582 046	0.164 554 068 128
252	2.710 153 462 785	4.412 645 922 929	0.683 262 056 637	0.311 022 140 748	0.164 554 280 867
444	2.710 174 411 985	4.412 686 908 807	0.683 261 807 233	0.311 021 590 292	0.164 548 040 929
1078	2.710 178 102 456	4.412 694 151 691	0.683 261 769 228	0.311 021 505 708	0.164 552 263 436
2856	2.710 178 273 496	4.412 694 488 233	0.683 261 767 691	0.311 021 502 230	0.164 552 859 097
4389	2.710 178 277 471	4.412 694 496 073	0.683 261 767 658	0.311 021 502 223	0.164 552 855 340
	$\langle \delta(r_{12}) \rangle$	$\langle \hat{T} \rangle$	$\langle \hat{V} \rangle$	ν_{21}	ν_{31}
22	0.002 895 756 698	0.527 679 151 455	-1.055 358 302 896	0.428 970 907 817	-0.992 389 671 789
95	0.002 756 352 077	0.527 750 090 407	-1.055 500 180 824	0.483 411 356 285	-0.999 711 128 276
161	0.002 745 511 476	0.527 750 868 154	-1.055 501 736 318	0.491 184 379 798	-0.999 973 141 163
252	0.002 741 380 780	0.527 750 986 223	-1.055 501 972 525	0.495 040 826 429	-1.000 016 451 639
444	0.002 739 160 227	0.527 751 012 092	-1.055 502 024 174	0.497 749 642 666	-0.999 812 228 789
1078	0.002 738 184 681	0.527 751 016 369	-1.055 502 032 747	0.499 417 108 213	-0.999 962 220 715
2856	0.002 738 016 013	0.527 751 016 540	-1.055 502 033 081	0.499 886 594 672	-0.999 999 970 351
4389	0.002 738 000 445	0.527 751 016 543	-1.055 502 033 087	0.499 946 268 831	-0.999 999 984 930

TABLE XI: Values of various expectation values for helium (He) with increasing basis set size for the Fully Correlated (FC) implementation.

No. terms	$\langle r_1 \rangle$	$\langle r_{12} \rangle$	$\left\langle \frac{1}{r_1} \right\rangle$	$\left\langle \frac{1}{r_{12}} \right\rangle$	$\langle \delta(r_1) \rangle$
22	0.929 422 140 200	1.421 970 897 235	1.688 317 024 041	0.945 838 029 675	1.810 504 584 655
95	0.929 471 891 736	1.422 069 471 064	1.688 316 805 600	0.945 818 598 472	1.810 397 917 976
161	0.929 472 230 470	1.422 070 130 641	1.688 316 801 722	0.945 818 472 100	1.810 416 192 514
252	0.929 472 281 993	1.422 070 230 595	1.688 316 800 912	0.945 818 453 341	1.810 423 547 641
444	0.929 472 292 452	1.422 070 250 919	1.688 316 800 792	0.945 818 449 575	1.810 426 446 718
1078	0.929 472 294 804	1.422 070 255 432	1.688 316 800 716	0.945 818 448 821	1.810 429 028 568
2856	0.929 472 294 876	1.422 070 255 563	1.688 316 800 717	0.945 818 448 801	1.810 429 296 331
4389	0.929 472 294 873	1.422 070 255 564	1.688 316 800 717	0.945 818 448 800	1.810 429 310 048
	$\langle \delta(r_{12}) \rangle$	$\left\langle \hat{T} \right\rangle$	$\left\langle \hat{V} \right\rangle$	ν_{21}	ν_{31}
22	0.107 243 763 234	2.903 715 033 297	-5.807 430 066 491	0.464 638 465 675	-2.000 293 015 431
95	0.106 409 227 098	2.903 724 311 855	-5.807 448 623 930	0.494 755 838 500	-1.999 799 397 169
161	0.106 367 384 264	2.903 724 367 396	-5.807 448 734 788	0.497 625 423 240	-1.999 890 310 366
252	0.106 353 955 189	2.903 724 375 093	-5.807 448 750 307	0.498 829 216 122	-1.999 939 779 403
444	0.106 347 374 019	2.903 724 376 815	-5.807 448 753 595	0.499 618 061 297	-1.999 962 298 408
1078	0.106 345 590 174	2.903 724 377 019	-5.807 448 754 046	0.499 933 901 477	-1.999 993 988 891
2856	0.106 345 396 399	2.903 724 377 031	-5.807 448 754 034	0.499 987 099 738	-1.999 999 171 813
4389	0.106 345 378 478	2.903 724 377 034	-5.807 448 754 068	0.499 993 950 385	-1.999 999 912 034

TABLE XII: Values of various expectation values for the lithium ion (Li^+) with increasing basis set size for the Fully Correlated (FC) implementation.

No. terms	$\langle r_1 \rangle$	$\langle r_{12} \rangle$	$\left\langle \frac{1}{r_1} \right\rangle$	$\left\langle \frac{1}{r_{12}} \right\rangle$	$\langle \delta(r_1) \rangle$
22	0.572 764 645 677	0.862 295 160 247	2.687 923 765 409	1.567 736 134 034	6.851 681 852 646
95	0.572 774 076 123	0.862 315 228 788	2.687 924 395 643	1.567 719 677 983	6.851 909 183 342
161	0.572 774 138 564	0.862 315 353 042	2.687 924 397 305	1.567 719 576 826	6.851 971 182 514
252	0.572 774 147 766	0.862 315 371 146	2.687 924 397 391	1.567 719 562 458	6.851 993 762 995
444	0.572 774 149 575	0.862 315 374 699	2.687 924 397 431	1.567 719 559 608	6.852 003 996 753
1078	0.572 774 149 964	0.862 315 375 441	2.687 924 397 409	1.567 719 559 148	6.852 008 805 199
2856	0.572 774 149 971	0.862 315 375 456	2.687 924 397 407	1.567 719 559 140	6.852 009 386 991
4389	0.572 774 149 971	0.862 315 375 455	2.687 924 397 410	1.567 719 559 138	6.852 009 176 559
	$\langle \delta(r_{12}) \rangle$	$\left\langle \hat{T} \right\rangle$	$\left\langle \hat{V} \right\rangle$	ν_{21}	ν_{31}
22	0.536 388 625 955	7.279 903 228 954	-14.559 806 458 424	0.467 031 232 384	-2.999 873 366 940
95	0.533 894 140 851	7.279 913 347 880	-14.559 826 695 874	0.495 499 590 111	-2.999 739 003 087
161	0.533 780 075 034	7.279 913 403 591	-14.559 826 807 009	0.498 009 466 056	-2.999 867 234 692
252	0.533 744 376 699	7.279 913 410 916	-14.559 826 821 891	0.499 204 582 959	-2.999 931 109 990
444	0.533 726 633 105	7.279 913 412 510	-14.559 826 824 982	0.499 739 949 218	-2.999 969 594 068
1078	0.533 723 034 182	7.279 913 412 643	-14.559 826 825 306	0.499 951 367 164	-2.999 994 440 414
2856	0.533 722 804 797	7.279 913 412 640	-14.559 826 825 305	0.499 970 689 552	-2.999 999 482 028
4389	0.533 722 796 738	7.279 913 412 656	-14.559 826 825 322	0.499 996 994 732	-2.999 999 927 605

II. HARTREE FOCK (HF) DATA

The HF energies and expectation values using a 20-term wavefunction (published previously in [1]) are reported in the main text. The optimised non-linear variational parameter and the coefficients for each HF wavefunction to 32 digits, along with the Hartree Fock energy convergence data, can be downloaded from the following URL:

`http://rsta.royalsocietypublishing.org/content/roypta/suppl/2018/01/24/rsta.2017.0153.DC1/rsta20170153supp1.pdf`

Additionally, a Maple script containing all the HF wave functions can be accessed via the Dryad Digital Repository (`http://dx.doi.org/10.5061/dryad.fr34t`).

III. INTRACULE AND COULOMB HOLE DATA

A complete data set of intracule and Coulomb hole data for each system (i.e. Z_C, H^-, He and Li^+) associated with this paper is available from the Dryad Digital Repository (`http://dx.doi.org/10.5061/dryad.r60sj21`).

---

[1] King AW, Baskerville AL, Cox H. 2018 Hartree-Fock implementation using a Laguerre-based wave function for the ground state and correlation energies of two-electron. *Phil. Trans. R. Soc. A.* **376**, 20170153. (doi:`http://dx.doi.org/10.1098/rsta.2017.0153`)
